# A Mobile Health App to Improve HIV Medication Adherence: Protocol for a Pilot Randomized Controlled Trial

**DOI:** 10.2196/15356

**Published:** 2019-11-13

**Authors:** Susan Ramsey, Evan Ames, Julia Uber, Samia Habib, Seth Clark

**Affiliations:** 1 The Warren Alpert Medical School of Brown University Providence, RI United States; 2 Rhode Island Hospital Providence, RI United States

**Keywords:** HIV, medication adherence, mobile health

## Abstract

**Background:**

Adherence to antiretroviral therapy (ART) is essential for allowing persons living with HIV to live longer, healthier lives. However, a large portion of this population has suboptimal adherence and are not virally suppressed. Conventional interventions aimed at improving ART adherence lack portability and scalability, and improvements in adherence are not often sustained. Mobile health (mHealth) ART interventions offer a low-cost and accessible method of improving adherence, but many have limited functionality and do not offer comprehensive support. The combination of an mHealth intervention with a face-to-face adherence intervention and interactive health coaching feature may offer sufficient support in a manner that is sensitive to resource limitations that are often found in HIV treatment settings. This paper details the protocol of a study designed to evaluate the potential of an enhanced mHealth intervention for improving ART adherence.

**Objective:**

The primary objective of this study is to assess the feasibility and acceptability of the Fitbit Plus app enhanced with a face-to-face LifeSteps session (Fitbit Plus condition) for improving ART adherence. In addition, we will determine the preliminary efficacy of the intervention by calculating treatment effect sizes.

**Methods:**

This study will be conducted in 2 phases. The intervention will be developed and piloted with a small group of participants during phase 1. Pilot participants will provide feedback that will be used to refine the intervention for phase 2. In phase 2, a preliminary randomized controlled trial (RCT) comparing Fitbit Plus with a condition that approximates the standard of care (SOC) will be conducted with 60 persons living with HIV. Interviews will be conducted with RCT participants at baseline, and follow-up interviews will be conducted at 1, 3, 6, and 12 months. ART adherence is the primary outcome and will be monitored throughout the study via electronic pill boxes. Effect sizes will be generated using a fractional logit model estimated by generalized estimating equations.

**Results:**

Phase 1 of this trial is complete; data collection for phase 2 is ongoing. Follow-ups with enrolled participants will conclude in January 2020.

**Conclusions:**

This study will contribute to the literature on ART adherence and may produce an efficacious intervention. Owing to a small sample size, there may be insufficient power to detect statistically significant differences between Fitbit Plus and SOC. However, if Fitbit Plus is found to be acceptable and feasible and yields promising effect size estimates, this pilot study could serve as the foundation for a larger, fully powered trial of Fitbit Plus.

**Trial Registration:**

ClinicalTrials.gov NCT02676128; https://clinicaltrials.gov/ct2/show/NCT02676128

**International Registered Report Identifier (IRRID):**

DERR1-10.2196/15356

## Introduction

### Background

The Centers for Disease Control and Prevention estimates that 1.1 million people living in the United States are infected with HIV [1], with an estimated 38,739 new HIV diagnoses in 2017 alone [2]. Antiretroviral therapy (ART) is highly effective and allows persons living with HIV (PLWH) to live longer, healthier lives [3,4]. Despite the effectiveness of ART, only 60% of PLWH living in the United States achieve viral suppression [5]. Medication nonadherence is a significant contributor to unsuccessful viral suppression; a meta-analysis found that only an estimated 59% of participants in North American studies were adherent at the commonly accepted minimal threshold for successful viral suppression of 90% [6]. Although newer medications can produce viral suppression at lower levels of adherence, relatively high adherence is still necessary to avoid disease progression and shortened lifespan [7-9].

Given the significant public health problem presented by poor adherence to ART, a great deal of research has been devoted to improving adherence. Interventions have been developed to address poor ART adherence, with most studies demonstrating some degree of success [10]. LifeSteps, one of the early interventions in this area, is a single session intervention that incorporates motivational interviewing, cognitive-behavioral skills, and problem-solving components and is grounded in the Information-Motivation-Behavioral Skills Model of ART adherence [11-13]. It was found to improve adherence more rapidly when compared with a self-monitoring condition [12] and has been used in conjunction with cognitive behavioral therapy (CBT) interventions to improve ART adherence and reduce depressive symptoms in PLWH [11,14,15]. A noted shortcoming of ART adherence interventions is the tendency of their treatment effects to diminish over time [10]; most real-world clinical settings lack the resources necessary to sustain any adherence improvements that are achieved [10].

To address concerns of portability and sustainability of improvements from traditional ART adherence interventions, effort has also been directed at the development of mobile health (mHealth) ART adherence interventions. mHealth ART interventions allow greater accessibility than clinical settings and provide a low-cost option for continued intervention. To date, the bulk of the research in this area has focused on text message–based ART adherence interventions. A meta-analysis of text messaging interventions [16] found fairly modest but significant support for these interventions. However, most of the interventions examined were of short duration, which is noteworthy given the well-known challenges of habituation and response fatigue with these types of interventions.

Currently, there are hundreds of HIV-related mHealth apps marketed for PLWH on either Android or Apple platforms. Fortunately, a number of mHealth apps for PLWH have been found to be effective, as well as feasible and acceptable [17]. However, most current mHealth interventions have limited functionality, such as only offering medication reminders [17]. Another review highlighted key content areas that are not currently addressed by existing apps: resources regarding psychological and emotional support and components that enhance linkage to treatment providers [18]. Therefore, more comprehensive mHealth interventions, which address multiple self-management needs of PLWH, are needed.

Evidence suggests that treatment supporters result in better adherence to ART [19]. Examples of treatment supporters include peer support sessions, home visits by nurses or counselors, case management, and provision of training in treatment support to a friend or family member of the patient. The importance of this type of adherence support has been found in other reviews as well [20,21].

Health coaches represent one type of treatment supporter. Health coaching has a patient-centered focus, involving patients in the process of goal setting [22]. Health coaches assist the patient in achieving a greater understanding of the patient’s medical condition and encourage patient accountability [22]. Reviews of the general literature regarding health coaching conclude that the approach shows great promise for improving health outcomes [23,24].

### Objective

One particular smartphone app that has shown promise in improving ART adherence incorporated personalized health-related information with medication reminders [25]. This paper outlines the protocol behind a study designed to extend and enhance this previous work. These enhancements include a face-to-face adherence intervention and an interactive health coaching feature, which were selected to address barriers and limitations of typical mobile adherence aids. At the conclusion of the study, we will be able to determine the feasibility, acceptability, and preliminary efficacy of this intervention.

## Methods

### Overview

To assess the feasibility, acceptability, and preliminary efficacy of the study intervention, we will develop and test the intervention through 2 phases. In phase 1, we will pilot and refine our face-to-face intervention and the use of our smartphone app (Fitbit Plus). In phase 2, we will conduct a randomized controlled trial (RCT) of 60 PLWH to examine the effect of the face-to-face intervention followed by Fitbit Plus (Fitbit Plus condition) compared with the face-to-face intervention alone, which approximates standard of care (SOC condition) in most HIV treatment settings. Participants in the RCT will complete a baseline interview and follow-up interviews at 1, 3, 6, and 12 months. The primary outcome will be ART adherence based on electronic pill box (EPB) data.

### Participants and Recruitment

A total of 80 PLWH (n=20 in phase 1 and n=60 in phase 2) will be recruited from the Northeast region of the United States via community advertisement and in-person recruitment at an outpatient HIV treatment center. Persons will be eligible for the study if they are at least 18 years of age, have been prescribed ART, are living with HIV, have had a detectable viral load (>20 copies/mL) within the past 6 months, report less than 100% self-reported medication adherence, and have a smartphone that is compatible with Fitbit Plus. Persons living with physical or cognitive impairment that could impede completion of the intervention or jeopardize informed consent, active psychosis, or who are not fluent in English will be excluded from participation. This study has been reviewed and approved by the first author’s institutional review board.

After receiving a description of the study from research staff, anyone interested in participating will be asked to complete a screening interview. The screening interview, which will include a review of medical information, will assess eligibility and will be used to ensure no exclusion criteria are met. Demographic information, including age, gender, time since HIV diagnosis, mode of HIV transmission, and type of cell phone used will be collected as a part of the screening interview. For screening purposes, a single-item visual analog scale [26] will be used to measure ART adherence for the past month. In this measure, respondents are asked to rate percentage of prescribed doses taken in the past month on a visual analog scale ranging from 0% to 100%. We will confirm the absence of cognitive impairment using the University of California, San Diego Brief Assessment of Capacity to Consent measure [27] and the absence of active psychosis using the Mini-International Neuropsychiatric Interview (MINI) [28], with positive responses to the MINI further queried by doctoral-level study staff. Written informed consent will be obtained from those who are eligible; this will be obtained at the time of the screening interview for those who are screened in person or during the first in-person visit for those who are screened over the phone.

### Phase 1: Development and Piloting Phase

#### Procedures

During phase 1, we will test the face-to-face adherence intervention followed by the use of Fitbit Plus with 5 to 10 PLWH. In-depth interviews will be conducted with these pilot participants after they have used Fitbit Plus for 3 months, in addition to brief phone interviews after 1 month of Fitbit Plus use, to gain an understanding of the strengths and limitations of the app as well as to garner feedback regarding the face-to-face session content. This feedback will guide refinement, and this process will then be repeated with another 5 to 10 PLWH. To enhance follow-up rates, participants in phase 1 will be paid US $50 for completing the postintervention interview. The data collected from these participants regarding feasibility, acceptability, and barriers or challenges that would limit effectiveness will guide modifications to the intervention.

#### Face-to-Face Antiretroviral Therapy Adherence Intervention

Our single session face-to-face ART adherence intervention will be based on the LifeSteps intervention [11,12,14,15]. Consistent with the literature regarding efficacious adherence interventions [21,29], LifeSteps combines brief motivational interviewing, CBT, and problem-solving skills and is grounded in the IMB Skills Model of ART adherence [13]. During the development phase of the study, Fitbit Plus orientation material will be added to the face-to-face intervention that will be delivered to pilot participants and to RCT participants assigned to the Fitbit Plus condition. The introduction to Fitbit Plus will include downloading the app to the participant’s phone, entering the participant’s ART medication schedule, and assisting the participant in formulating an ART adherence goal, which will be entered into the app so that both the patient and health coach can track progress toward the goal. Participants who do not have password protection enabled on their phones will be encouraged to activate this feature to protect the privacy of their information. During the orientation to Fitbit Plus, we will highlight that the interactive coaching feature is not intended for use in medical or other emergencies and that side effect and other medical questions should be directed to clinic treatment providers. Pilot participant feedback will guide refinement of the face-to-face intervention, including the Fitbit Plus orientation content, during phase 1 of the study.

#### The Mobile Health Antiretroviral Therapy Adherence App (Fitbit Plus)

An earlier version of Fitbit Plus (previously known as Twine) was found to show promise in improving ART adherence [25]. Fitbit Plus is fully Health Insurance Portability and Accountability Act compliant. The participant version of Fitbit Plus runs on an iPhone with iOS version 9.0+ or an Android phone with version 4.4+. The vast majority (94%) of smartphones in the United States use a platform (iOS or Android) that is compatible with Fitbit Plus [30]. Therefore, the proposed intervention could be disseminated readily. In addition to running on smartphones, the health coach version also runs on a Chrome, Firefox, or Safari Web browser or an iPad with iOS version 9.0+. The Fitbit Plus app that is compatible with any medication, includes medication reminders and tracking, and secure 2-way messaging. Fitbit Plus will generate a push notification at the time ART dosing should occur each day. After each push reminder, participants are prompted to click *yes* or *no* that they have taken their medication. Responding to the prompt requires only the single click of a text box, minimizing respondent burden.

On their “dashboards,” health coaches will be able to monitor participants’ adherence to ART in real time and identify participants whose adherence falls below optimal levels and may be in need of support, in addition to responding to participant requests for support or information. There is general support in the extant ART adherence literature for adherence monitoring and feedback [31,32]. Health coaches will support ART adherence through interactive secure messaging. Health coaches will provide support, encouragement, and resources, including links and attachments, via the messaging feature. At a minimum, health coaches will message participants at least weekly. In the development phase of the project, we will develop content that health coaches can send via the app to address commonly encountered situations that impede ART adherence (eg, stress/anxiety, depressive symptoms, substance use, and treatment fatigue). Coaching materials and strategies will also be refined based on pilot participant feedback. Fitbit Plus will allow us to examine utilization data for both the adherence tracking and interactive features.

#### Training and Supervision of Health Coaches

Health coaches will receive training and supervision in the face-to-face intervention and the interactive coaching component of the mHealth ART adherence app from the first author who has extensive experience in the training, supervision, and delivery of interventions grounded in motivational interviewing and CBT. Training will include didactics, role-playing, and review of audiotaped sessions. Weekly supervision will be held to ensure competent and standardized delivery. All face-to-face sessions will be audiotaped, and the content of the interactive coaching messages delivered via Fitbit Plus will be logged. A randomly selected 30% (18/60) of face-to-face session recordings and Fitbit Plus coaching messages will be rated for health coach competence and adherence to intervention protocol.

### Phase 2: Randomized Controlled Trial Phase

#### Procedures

After informed consent has been obtained, participants will complete a baseline interview (see Table 1 for timeline of procedures). EPBs will be distributed at the conclusion of the baseline interview, with instructions to begin storing their ART medication in the EPB and to continue to adhere to their regimen as they normally would. Adherence will then be monitored for a 2-week period to establish a baseline adherence level. Participants will be randomized to Fitbit Plus or SOC using urn randomization [33] to ensure that the 2 groups are comparable on important prognostic variables (number of years on ART, percent self-reported adherence on the visual analog scale [26], level of substance use reported on the Timeline Followback (TLFB) interview [34], and level of depressive symptoms reported on the Centers for Epidemiologic Studies-Depression Scale [35]). Participants assigned to the Fitbit Plus condition will receive the face-to-face intervention followed by 12 months of access to Fitbit Plus, whereas the SOC condition will receive the face-to-face session alone. Participants will be informed whether or not they are receiving the app during the face-to-face session, and only Fitbit Plus participants will receive an introduction to the app. Follow-up interviews will be conducted 1, 3, 6, and 12 months after the baseline interview. Participants in phase 2 will be compensated US $25 for completion of the baseline interview and US $30, US $35, US $40, and US $50, respectively, for completion of the 1-, 3-, 6-, and 12-month follow-ups.

#### Measures

All data collection will be captured with a secure electronic data capture system developed by Vanderbilt University, REDCap. Paper versions of the assessments can be provided upon request but then will be entered by research staff into REDCap (see Table 2 for schedule of assessments).

**Table 1:** Timing of participant enrollment, receipt of interventions, and assessment activities during phase 2.

Timepoint		Enrollment (–t1)	Baseline (0)	Intervention start (+2 weeks)	Post allocation			
					+1 month	+3 months	+6 months	+12 months
**Enrollment**								
	Eligibility screen	✓^a^	—^b^	—	—	—	—	—
	Informed consent	✓	—	—	—	—	—	—
	Allocation	—	✓	—	—	—	—	—
**Interventions**								
	Fitbit Plus	—	—	✓	✓	✓	✓	✓
	Standard of care	—	—	✓	—	—	—	—
**Assessments**								
	Primary outcomes	—	✓	—	✓	✓	✓	✓
	Secondary outcomes	—	✓	—	✓	✓	✓	✓
	Other outcomes	—	✓	—	✓	✓	✓	✓

^a^Indicates study activity will occur at the corresponding time-point.

^b^Not applicable.

**Table 2:** Schedule of assessments.

Measures		Baseline	1-month follow-up	3-month follow-up	6-month follow-up	12-month follow-up
**ART^a^ adherence**						
	Electronic pill box data	✓^b^	✓	✓	✓	✓
	Adult AIDS Clinical Trials Group medication questionnaire	✓	✓	✓	✓	✓
	3-item ART adherence questions	✓	✓	✓	✓	✓
Viral load		✓	—^c^	—	✓	✓
**Potential treatment mechanisms**						
	LifeWindows information-motivation-behavioral skills ART questionnaire	✓	✓	✓	✓	✓
	HIV Treatment Adherence Self-Efficacy Scale	✓	✓	✓	✓	✓
**Potential moderators of treatment effects**						
	Timeline Followback	✓	✓	✓	✓	✓
	Center for Epidemiological Studies-Depression Scale	✓	✓	✓	✓	✓
	Fägerstrom Test for Nicotine Dependence	✓	✓	✓	✓	✓
**Treatment received**						
	Treatment services review	✓	✓	✓	✓	✓
	Use of medications	✓	✓	✓	✓	✓
**Feasibility and acceptability**						
	Fitbit Plus satisfaction questionnaire	—	—	—	✓	✓
	Semistructured interview	—	—	—	—	✓

^a^ART: antiretroviral therapy.

^b^✓ indicates measure will be administered at the corresponding time-point.

^c^Not applicable.

#### Antiretroviral Therapy Adherence

##### MEMS Cap

ART adherence will be measured via EPB throughout the course of the study. Participants will be asked to bring the EPB to each follow-up interview. At each follow-up interview, adherence data (dates and times of pill bottle openings) will be downloaded from the EPB and then returned to the participant for continued medication adherence monitoring. At the last study visit (12-month follow-up), EPBs will be collected from participants, and all data will be downloaded and then deleted from the device.

Although this is an objective measure of adherence, there are limitations that should be noted. As the data from most EPBs must be downloaded using a specific device, there is a potential for data loss if a participant never brings the EPB to a study visit or misplaces it entirely. We will attempt to mitigate this in the following ways: (1) by reminding participants to bring the cap to every visit and (2) having replacements available should a participant report misplacing their EPB. These strategies have successfully minimized data loss in previous longitudinal studies of medication adherence [36-39].

In addition, adherence rates may be artificially inflated as EPBs record the date and time every time it is opened. It does not differentiate purposes for opening (eg, taking medication vs refilling pill bottle). EPBs could also potentially underestimate adherence rates owing to situations in which participants open the device, take out multiple doses, and then do not open the device again for several days but still take their medication (eg, pocketing doses for weekend travel). At each study appointment, participants will be asked if they can recall any instances in which they (1) opened the EPB but did not take medication or (2) took their medication but did not open the EPB. This information will be added to the EPB data to create a self-report corrected version of the EPB data. As is typically done in ART adherence studies (eg, [40]), we will consider the self-report corrected EPB data to be the primary ART adherence data. However, we will also conduct our planned data analyses on the uncorrected EPB data to bolster our confidence in the findings.

#### Self-Reported Adherence

Self-reported adherence will be collected at baseline and follow-up interviews using the Adult AIDS Clinical Trials Group medication questionnaire that asks participants to report on the number of doses missed of their prescribed ART within the last 4 days [41]. We will also assess self-reported adherence with a 3-item ART adherence measure developed by Wilson and colleagues [42]. This brief measure has been found to have good psychometric properties and good construct validity when compared with electronic drug monitoring [42].

#### Viral Load

Viral load data will be collected at baseline and at the 6- and 12-month follow-up appointments. These data will be collected through a laboratory belonging to a hospital system in the Northeastern region of the United States. The laboratories use assays with sensitivity to detect viral load >20 copies/mL.

#### Potential Treatment Mechanisms

A total of 2 factors that may be mechanisms of successful treatment for improving ART adherence are motivation and self-efficacy. The LifeWindows IMB ART Adherence Questionnaire (LW-IMB-AAQ) [43] is designed to measure barriers to ART adherence that fall within the information, motivation, and behavioral skills areas. These areas are consistent with the IMB model of adherence [13]. Self-efficacy for adherence to HIV medications will be assessed using the HIV Treatment Adherence Self-Efficacy Scale (HIV-ASES), which has been shown to have robust internal consistency and reliability [44]. The HIV-ASES is a 12-item scale of patient confidence in their ability to carry out behaviors related to adhering to medication regimens. Responses range from 1 (“cannot do it at all”) to 10 (“completely certain can do it”). Item scores are averaged, with higher scores indicating higher adherence self-efficacy. The LW-IMB-AAQ and HIV-ASES will be administered at the baseline and follow-up interviews.

#### Potential Moderators of Treatment Effects

There are well established links between substance use, depressive symptoms, and poor ART adherence among PLWH [45-47]. Therefore, we will evaluate whether substance use and/or depressive symptoms moderate treatment effects. The TLFB interview will be administered to assess daily alcohol and drug use at baseline, as well as during the follow-up interviews. The TLFB interview is a calendar-assisted structured interview that provides a way to cue memory so that accurate recall of prior substance use is enhanced [48,49]. The TLFB interview has excellent reliability [50] and validity [48] and will provide data on the number of standard drinks consumed per day and types of drug classes used each day. At baseline, it will be administered to assess substance use during the 3 months before the interview. The Center for Epidemiological Studies-Depression Scale [35] will be used to measure level of depressive symptoms at baseline and follow-up interviews. This 20-item measure is widely used and has good sensitivity and specificity and high internal consistency [51]. Finally, nicotine dependence has also been associated with poor ART adherence [52,53]; thus, we will collect information about nicotine dependence during the baseline and follow-up interviews with the widely used Fägerstrom Test for Nicotine Dependence [54].

#### Treatment Received

The Treatment Services Review (TSR) [55] will be used to assess receipt of case management, psychiatric, substance use, and other treatment services. At baseline, participants will be asked about services received in the previous 3 months. At follow-up interviews, they will be asked about services received since the previous interview. We will modify the TSR to ask specifically about HIV-related services received, including medical treatment for any ART-related side effects or HIV-related sequelae. Participants will also be asked to provide information regarding the use of medications for medical, psychiatric, or substance use indications. At baseline, patients will be asked to provide information regarding medications used in the past 3 months. At each follow-up interview, they will be asked to provide information concerning use of medications since the previous interview.

#### Feasibility and Acceptability

At the conclusion of the study, we will compile a patient eligibility rate, enrollment refusal rate, rate of recruitment, and follow-up completion rate to evaluate the feasibility of conducting a subsequent larger scale study using this protocol. We will also compile a study dropout rate and intervention session completion rate as indices of acceptability. Intervention acceptability and feasibility will also be assessed by asking participants randomized to receive Fitbit Plus satisfaction questions at the 6- and 12-month follow-ups. These questions will allow us to collect participants’ overall opinions of the smartphone app and will allow us to observe whether satisfaction changes between 6 and 12 months. In addition, a semistructured interview will be completed with these participants at the 12-month follow-up to solicit additional information about the acceptability and feasibility of the intervention. More specifically, the semistructured interview will allow us to ask questions about different features of the app, what their favorite and least favorite aspects were, and if they have any recommendations for improvement.

### Planned Analyses

Tests of the effects of treatment on the primary outcome variable (percent ART adherence based on corrected EPB data at 1-, 3-, 6-, and 12-month follow-ups) will be conducted using a fractional logit model [56] estimated by generalized estimating equations (GEEs) [57-59]. GEE is a quasi-likelihood estimation method of repeated measures analysis that allows for the inclusion of both categorical and continuous independent variables and for appropriate modeling of covariance structures when outcomes are correlated across time. The primary, between groups, independent variable in the above regression analysis is treatment group assignment. Variables measured at baseline will be examined using screening runs before primary analyses to see which of these baseline variables are most strongly associated with the primary outcome (adherence based on EPB data) in our sample. Those that show significant relationships with outcome will be entered as additional covariates in the primary analyses. The linear effect of time will also be included as a covariate in these analyses, as we assume that adherence rates will show a tendency to decrease over time. We will also test for nonlinear (ie, higher order) effects of time. Testing the time by group interaction will indicate the extent to which treatment differences are more or less pronounced closer in time to the intervention.

The same process described above for EPB adherence data will be employed to examine the impact of Fitbit Plus, relative to SOC, on the secondary measures of self-reported adherence percentage and viral load (dichotomized). Changes in viral load test assays have created inconsistencies in the literature in regard to level of viral load that is detectable. To be able to compare our results with the extant literature, we will dichotomize viral load at the 3 most commonly used levels (20, 50, and 200 copies/mL) and examine intervention effects using each of these levels. We will then explore potential mediators (motivation and self-efficacy) of the intervention effect on HIV medication adherence. This will be done using 2 common approaches to evaluating mediation mechanisms; Baron and Kenny’s approach [60] and structural equation modeling [61,62].

In addition to the analyses that address the specific aims of the study, our data set will allow us to perform exploratory analyses that could enhance our understanding of ART adherence and may suggest avenues for future research. We will examine the temporal association between alcohol and drug use (TLFB) and missed ART doses. Using hierarchical linear models, we will regress daily ART adherence (based on EPB data) onto daily substance use. This will allow for the calculation of odds ratios that reflect the extent to which alcohol and drug use on a given day are associated with ART adherence on that day, relative to days in which participants refrained from using substances. Given that ART adherence will be examined dichotomously for these analyses, a logistic regression model will be specified in all analyses. More fine grained analyses will allow us to examine whether certain classes of drugs or level of alcohol consumption are temporally associated with missed ART doses.

## Results

Funding for this project began in July 2015. Recruitment for phase 1 of this trial began in February 2016 and was completed in October 2016. Recruitment for phase 2 of this trial began in November 2016, and data collection is ongoing. A total of 53 participants have been enrolled in phase 2 and were randomized to treatment condition. At this time, the follow-up rate at the 12-month follow-up is 90%. It is anticipated that the final follow-up appointment will occur in January 2020. Formal analysis of deidentified data will proceed after the conclusion of data collection.

## Discussion

There is a pressing need for more effective and accessible interventions for improving ART adherence given the high number of PLWH who are nonadherent and have not achieved viral suppression [5,6]. This paper provides an outline of a protocol for a research study that aims to evaluate the acceptability, feasibility, and preliminary efficacy of an enhanced smartphone app intervention for improving adherence to ART among PLWH. Over a 2-phase design, this study will refine the Fitbit Plus intervention and compare this with an SOC condition in a preliminary RCT. The Fitbit Plus intervention is designed to overcome barriers and limitations of traditional mHealth apps for ART adherence [17]. Given the extant literature, we anticipate that Fitbit Plus will be feasible and acceptable and will improve ART adherence [31,32].

Although this study promises to contribute to the literature on ART adherence, there are also important limitations that need to be considered. As this is a pilot study, the sample size that will be recruited is sufficient for establishing acceptability and feasibility of the intervention. However, we may not be adequately powered to detect statistically significant differences between groups. This is especially true regarding analyses containing higher order effects and multiple predictors. Rather, our goal is to yield a stable effect size estimate of treatment effects that can be used to justify a larger scale RCT. In addition, persons younger than 18 years will be excluded from participation, limiting the generalizability of study findings. ART adherence among adolescents living with HIV is also a major public health concern [63]. Should this intervention be found efficacious within the current population, it will need to be tested among teenagers and young adults to determine if modification is required.

Despite the limitations outlined above, this study will contribute to the literature in very important ways. First, we will gain a preliminary understanding of whether the inclusion of an in-person adherence intervention session and a smartphone app with interactive health coaching improves ART adherence. In addition, we will also be able to explore whether Fitbit Plus helps to sustain treatment effects over a 12-month period. A further contribution of the proposed study is the examination of measures of putative mechanisms, grounded in the IMB model [13,64], through which Fitbit Plus may impact adherence. These analyses hold the potential to increase our understanding of the adherence intervention treatment mechanisms. Finally, our battery of assessment measures will allow us to conduct very interesting exploratory analyses. For example, we will be able to examine the temporal association between substance use and missed ART doses using data collected for each day during the course of the study. In summary, this study has the potential to produce an efficacious intervention for improving adherence to ART.

## Abbreviations

ART: antiretroviral therapy
CBT: cognitive behavioral therapy
EPB: electronic pill box
GEE: generalized estimating equation
HIV-ASES: HIV Treatment Adherence Self-Efficacy Scale
IMB: information-motivation-behavioral
LW-IMB-AAQ: LifeWindows IMB ART Adherence Questionnaire
mHealth: mobile health
MINI: Mini-International Neuropsychiatric Interview
PLWH: persons living with HIV
RCT: randomized controlled trial
SOC: standard of care
TLFB: Timeline Followback
TSR: Treatment Services Review
